# In support of early career researchers

**DOI:** 10.1038/s41467-021-23455-8

**Published:** 2021-05-13

## Abstract

Opportunities for early career researchers (ECRs) to engage with the peer review and publication process can be few and far between. Last year, we launched a pilot support programme to introduce ECRs to peer review.

Early career researchers (ECRs)—PhD students, post-docs as well as recently-appointed principal investigators—constitute a large fraction of the scientific community, however they are often excluded from direct interaction with the publishing process, particularly with regards to reviewing the work of others. Frequently, there is no formal training available on how to become a fair and constructive peer reviewer. The PRE-review Open Reviewers pilot programme aimed to address this gap by providing formal training material to ECR mentees and journal editor mentors who then engaged in reviewing preprints in pairs. But usually, if they are lucky, ECRs can learn on the job by performing a review under the supervision of more experienced colleagues.

“82% would recommend the programme to another researcher”

Even in that case, it is not unusual for the ECR to not receive credit for the work performed as a reviewer. To address this issue, in 2020, the Nature Reviews journals initiated a programme to encourage reviewers to enroll and mentor an ECR from their group through the peer review of an article, while providing at the same time a framework to formally recognise the work of the mentee.

However, for the most part, the situation is frustrating for the ECRs, who feel left out of the publishing system. This cohort of investigators will lead the future of research but currently the peer review process is not benefiting from their diverse contributions as much as it could to shape advances within their fields.

In an effort to make peer review more inclusive for those investigators, in 2020 we piloted a support programme for ECRs in collaboration with Sense about Science, a UK-based charity that “champions the public interest in sound science and ensures evidence is recognised in public life and policy making”. The programme started with two webinars; the first one delved into the need for peer review, including its importance for research reporting and scientific progress, while the second discussed the specifics of editorial processes and gave practical guidance on how to be a fair reviewer and provide constructive suggestions aimed at improving the technical soundness and rigour of the conclusions presented.

The webinars were attended by about 200 ECRs from the Voice of Young Science network. About 30 of those investigators were selected to participate in a hands-on phase in which they received the direct support of our editors. Each participant was paired with one of the editors at *Nature Communications*, based on their field of expertise. The editor then invited the ECR to peer review a relevant article submitted to the journal, in addition to the usual set of reviewers sought for each paper. This was an opportunity for the participants to put into practice the advice received during the webinars. Upon return of the reviewer report, the ECR received feedback from the editor on the quality and usefulness of their comments, and further suggestions on how to write a high-quality reviewer report.

A survey of the participants showed very positive results: 73% said that the feedback received from the editor had been helpful or very helpful, 91% agreed that the programme gave them more confidence to review papers, and 82% would recommend the programme to another researcher. Many commented that the programme not only gave them the toolkit for peer reviewing, but also more generally supported their confidence and professional development.

The ECRs particularly valued the opportunity to review the work of others in a safe space, and would have been keen to review more papers. We identified areas of improvement in the programme that will ensure a better expertise match of the participants to relevant papers.

The small-scale pilot has been useful to test the logistics of the programme, and this year we want to offer support to as many ECRs as we can, focusing in particular on those who otherwise have more limited access to journals and editors—for example, if their institute is not located where in-person conferences usually take place.

Having built relationships with this cohort of new reviewers, we look forward to meeting and engaging with many more future and diverse research leaders this year. We are starting enrollment now and the programme will start in June—if interested, please apply!

